# Identification of genomic signatures in bone marrow associated with clinical response of CD19 CAR T-cell therapy

**DOI:** 10.1038/s41598-022-06830-3

**Published:** 2022-02-18

**Authors:** Lipei Shao, Avinash Iyer, Yingdong Zhao, Rob Somerville, Sandhya Panch, Alejandra Pelayo, David F. Stroncek, Ping Jin

**Affiliations:** 1 Center for Cellular Engineering, Department of Transfusion Medicine and Cellular Engineering, NIH Clinical Center, Bethesda, MD USA; 2 Biometric Research Program, Division of Cancer Treatment and Diagnosis, National Cancer Institute, Rockville, MD USA

**Keywords:** Predictive markers, Immunotherapy

## Abstract

CD19 CAR T-cell immunotherapy is a breakthrough treatment for B cell malignancies, but relapse and lack of response remain a challenge. The bone marrow microenvironment is a key factor in therapy resistance, however, little research has been reported concerning the relationship between transcriptomic profile of bone marrow prior to lymphodepleting preconditioning and clinical response following CD19 CAR T-cell therapy. Here, we applied comprehensive bioinformatic methods (PCA, GO, GSEA, GSVA, PAM-tools) to identify clinical CD19 CAR T-cell remission-related genomic signatures. In patients achieving a complete response (CR) transcriptomic profiles of bone marrow prior to lymphodepletion showed genes mainly involved in T cell activation. The bone marrow of CR patients also showed a higher activity in early T cell function, chemokine, and interleukin signaling pathways. However, non-responding patients showed higher activity in cell cycle checkpoint pathways. In addition, a 14-gene signature was identified as a remission-marker. Our study indicated the indexes of the bone marrow microenvironment have a close relationship with clinical remission. Enhancing T cell activation pathways (chemokine, interleukin, etc.) in the bone marrow before CAR T-cell infusion may create a pro-inflammatory environment which improves the efficacy of CAR T-cell therapy.

## Introduction

Chimeric antigen receptor-engineered T cells (CAR T-cells) are a new immunotherapy and they have emerged as an efficacious treatment of hematological tumors^1–3^. Multiple clinical trials have evaluated CAR T-cell therapy for B cell malignancies and have demonstrated promising outcomes by targeting CD19, CD22 or the combination of CD19 and CD22^4–6^. In fact, CD19 CAR T-cell therapy for acute lymphoblastic leukemia (ALL) in children and adults with relapsed and refractory disease have achieved remarkable efficacy with up to 90% complete response rate^4,7,8^. Despite the clinical success of anti-CD19 CAR T-cell therapy, there are still patients who do not go into remission or experience a disease relapse^9,10^. Furthermore, the determinants of a favorable clinical response to CAR T-cell therapy and disease resistance and relapse are largely unknown. Thus, it is important to identify biological markers associated with clinical response in order to enable clinicians to determine which patients will respond to CAR T-cell treatment in advance so that treatments can be adjusted for the maximization of therapeutic effects.

Preliminary studies have explored predictive markers related to clinical remission based on the function of T cells in final CAR T-cell products and patients’ baseline clinical status. For example, in vivo expansion of CD19 CAR T-cells and in vivo persistence of CD19 CAR T-cells has been associated better clinical response. Furthermore, an increased proportion of stem cell memory T cells (Tscm) in the final CAR T-cell products is a positive marker for CAR T-cell in vivo expansion and response, whereas a high frequency of effector memory T cells (Tem) negatively affects CAR T-cell proliferation and cytotoxicity^11,12^. However, few studies have investigated the relationship between clinical response and the bone marrow microenvironment prior to CAR T-cell therapy. The bone marrow microenvironment contains a cellular compartment (bone marrow mesenchymal stem and progenitor cells, adipocytes, immune cells, stromal cells) and a non-cellular compartment (growth factors, adhesion factors, and chemokines)^13^. It has been reported the proportion of CAR T-cells in bone marrow was higher in patients with complete remission compared to those with no response^14^, which suggests the microenvironment of bone marrow as a candidate to explore when investigating factors influencing CAR T-cell potency.

Public RNA-seq data are a rich resource for elucidating the mechanisms of human disease. Combining multiple individual datasets in one analysis increases the statistical power and makes it possible to gain additional insight on underlying biological mechanisms^15,16^. However, datasets from different research teams are not available in a consistent format in public transcriptome databases. Most teams directly uploaded the final gene expression matrix, while others uploaded raw sequencing data which needs an extra processing step using a series of bioinformatic pipelines to develop a gene expression matrix. The best way to resolve this issue is to develop a standardized bioinformatic pipeline and made it available for downstream analysis.

In this study, we evaluated 31 bone marrow samples obtained from patients with ALL from two centers before CD19 CAR T-cell therapy. We acquired their transcriptomic profiles based on an identical bioinformatic workflow and identified genomic signatures that could be used as predictors for clinical remission. All analyses were performed using well-established algorithms and are provided to facilitate future laboratory studies. In summary, we identified key bone marrow microenvironment-related expression signatures that are associated with clinical response in ALL patients treated with CD19 CAR T-cells.

## Methods

### Principal component analysis

Principal component analysis (PCA) was conducted using factoextra package in R environment (version 3.6.1). Custom code was uploaded into github public website (https://github.com/LIPEISHAO/RemissionMarker_project).

### Calculation of differentially expressed genes

The limma R package was used to generate p-values and fold change (FC) for each gene between samples with and without remission as a result to the treatment. This package takes the unnormalized, raw counts of the expression matrix and the corresponding metadata as inputs. The package then models the raw counts using normalization factors to account for the range of expression values. Next, the package estimates gene-wise variances and shrinks these estimates to model the counts. Finally, the package fits the negative binomial model to the dataset and perform Wald’s Test to calculate the *p* value or significance that a gene is differentially expressed. Genes with a *p*-value ≤ 0.01 and a │log_2_ (FC)│ ≥ log_2_(1.5) were identified as differentially expressed genes (Table S2).

### Gene ontology analysis

Using the R package, clusterProfiler (version 3.0.4)^17^, gene ontology (GO) analysis was performed on the dataset. GO analysis varies from GSEA as it utilizes a different annotation set and accounts for gene length bias in detection of over/ under representation of genes. With the hg37 annotation set, we performed enrichment analysis on our set of differentially expressed genes. We utilized log2(FC) and the *p*-value to determine significant genes for this analysis. Then we determined which GO terms were over or under-represented and visualized the data. We grouped the GO terms by biological process (BP), cellular component (CC), and molecular function (MF), and selected the significant, over-represented terms based on *p*-value < 0.05.

### Gene set analysis

Gene set enrichment analysis (GSEA) was performed in the R environment using the fgsea package based on the raw gene expression matrices. This software utilizes Log2(FC) and gene identities to determine pathways and their level of expression. The software then used the resulting pathways and the Log2(FC) to perform its analysis. The difference between this type of analysis is that it looks at genes in an entire set instead of individual genes. The GSEA works by first calculating an enrichment score (ES) that represents the amount a gene is overrepresented. Next, a *p*-value is determined by permutating the genes in the set. The pathways were organized based on *p*-value and NES score, and we selected significant pathways based on these criteria. Gene set variation analysis (GSVA), another R package was used to look at enrichment scores for custom pathways in order to check pathway activity specifically involved in T-cell function. *P*-value under 0.05 represents statistical significance.

### Prediction model construction

PAM approach was used to build a prediction model for remission^18^. PAM was proposed as a modification of the nearest-centroid method, called ‘‘nearest shrunken centroid.’’ This approach uses ‘‘de-noised’’ versions of the centroids as prototypes for each class. Discriminant score of predictive models is calculated as below:

$$ \begin{aligned} &\updelta _{{{\text{CR}}}} \left( {x*} \right) \, = \, \sum_{i} ({\text{x}}_{{\text{i}}} * - {\overline{\text{x}}}_{{{\text{iCR}}}}^{\prime } )^{2} {/}\left( {{\text{s}}_{{\text{i}}} + {\text{s}}_{0} } \right)^{2} - 2\log \left( {\pi_{{{\text{CR}}}} } \right) \\ &\updelta _{{{\text{NR}}}} \left( {x*} \right) \, = \, \sum_{i} ({\text{x}}_{{\text{i}}} * - {\overline{\text{x}}}_{{{\text{iNR}}}}^{\prime } )^{2} {/}\left( {{\text{s}}_{{\text{i}}} + {\text{s}}_{0} } \right)^{2} - 2\log \left( {\pi_{{{\text{NR}}}} } \right) \\ \end{aligned} $$

x_i_* is the log intensities of gene expression, ${\overline{\text{x}}}_{{{\text{iCR}}}}^{\prime }$ is the shrunken centroid of class CR for the *i*-th gene, ${\overline{\text{x}}}_{{{\text{iNR}}}}^{\prime }$ is the shrunken centroid of class NR for the *i*-th gene, s_i_ + s_0_ is the standard deviation for the *i*-th gene, π_CR_ is the prior probability for class CR, π_NR_ is the prior probability for class NR.

The signature gene, shrunken centroids and standard deviations are defined in Table 1. All process was finished in BRB_ArrayTools^19^. Leave-one-out cross-validation method was used to evaluate the model performance.

**Table 1:** Values of parameters in predictive model equation.

Gene symbol	Shrunken centroid in class CR	Shrunken centroid in class NR	Standard deviation S_i_ + S_0_
IGF2BP1	4.44	6.43	3.62
KCNK3	4.92	6.23	3.28
NKAIN4	5.25	6.62	3.66
GREB1	4.9	5.51	2.79
LAMB4	4.15	4.57	2.18
ADAMTS7	5.52	5.87	2.94
KIF26B	4.55	4.79	2.54
NETO1	5.32	5.6	3.47
TRIM9	5.57	5.69	2.21
RAB6C	4.7	4.84	2.8
TYRO3	5.25	5.36	2.31
KCNN1	5.3	5.46	3.62
HAP1	6.29	6.39	3.38
CCDC155	4.55	4.56	2.53

### Statement

All expression data were downloaded from public datasets and all methods were performed in this manuscript in accordance with the relevant guidelines and regulations.

## Results

### Data collection and processing prior to downstream analysis

We first collected related data from three known public datasets, including Gene Expression Omnibus (GEO), National Genomics Data Center (NGDC) and European Nucleotide Archives (ENA) using the key words “acute lymphoblastic leukemia”, “CD19”, and “immunotherapy”. The key words were imputed one at a time and in combination. Datasets were recruited when they included expression matrices and metadata describing clinical outcomes. In the end, only two datasets meet these requirements for downstream analysis^14,20^. All patients in this study were diagnosed with B-cell Acute lymphoblastic leukemia (B-ALL) and given CD19 CAR T-cell therapies. Of the 31 patients 22 achieved a clinical outcome of complete response (CR) while 9 showed no response (NR). One of the datasets, GSE153670, included patients enrolled in 3 multicenter clinical trials and it lacked information on age, gender, and cytotoxicity (Table S1).

**Figure 1:**
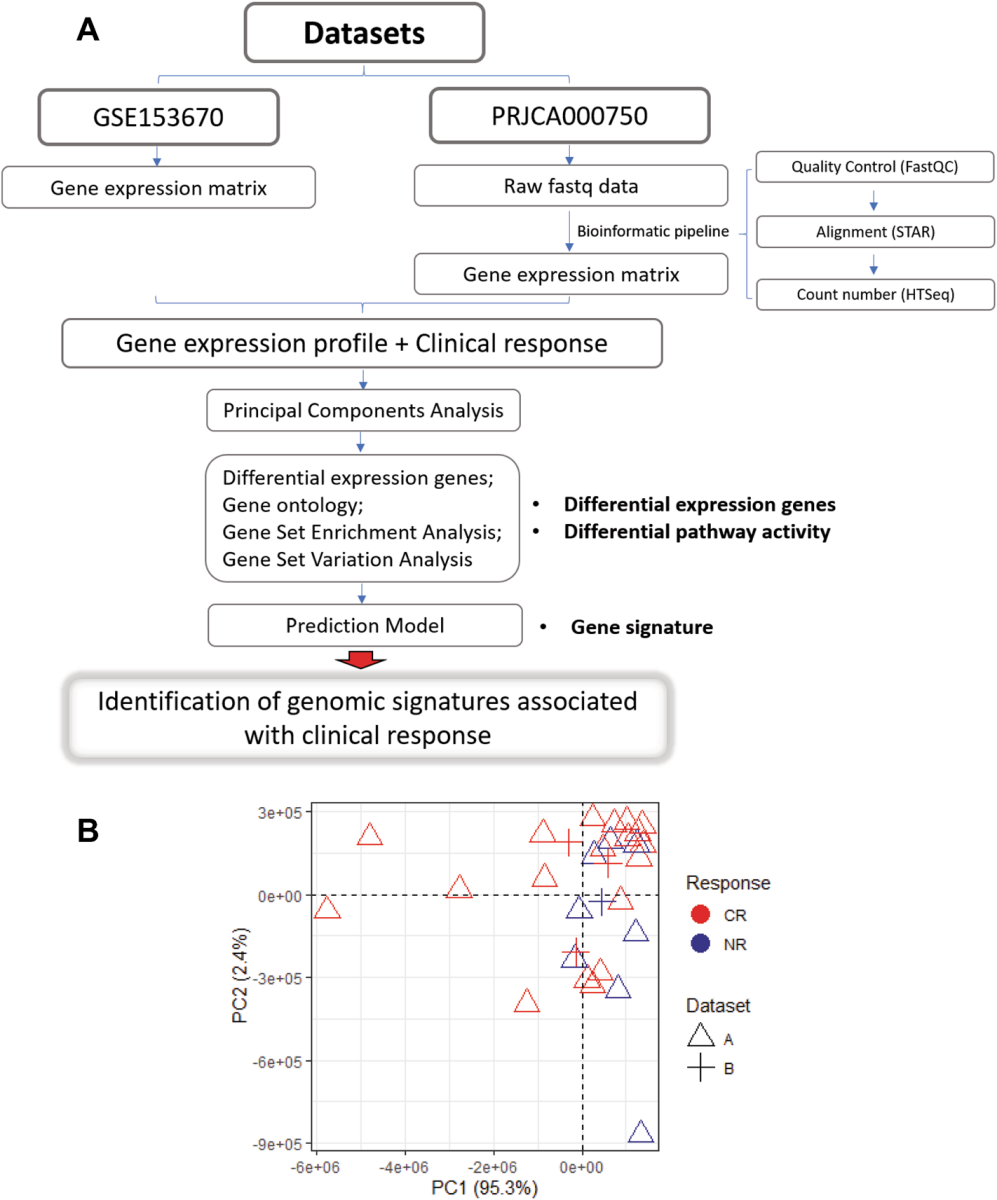
Schematic workflow of study. (**A**) Flowchart depicting the approach to identify the signatures; (**B**) two-dimensional scatter plot representing sample distribution according to the first two components obtained from principal component analysis (PCA) of the complete RNA-seq data. Dots are colored by response group, shaped by different datasets.

As shown in Fig. 1A, we developed a workflow to process these data and combined them in order to identify genomic signatures related to clinical outcomes. GSE153670 dataset provided a final gene expression matrix, while PRJCA000750 only uploaded raw fastq data. In order to resolve this inconsistency in the bioinformatic pipeline, we processed the fastq data with same software (FastQC, STAR, HTseq) used in GSE153670 dataset.

To determine if there was a batch effect that would hinder our ability to objectively analyze the data, we performed principal component analysis (PCA) on the combined dataset and found there are no obvious clustering of samples by dataset indicating that there was no batch effect among the datasets (Fig. 1B). This indicated that the combined expression data could be used for downstream analysis.

### Bone marrow genes upregulated in patients experiencing complete remission are mainly involved in T cell activation and differentiation

**Figure 2:**
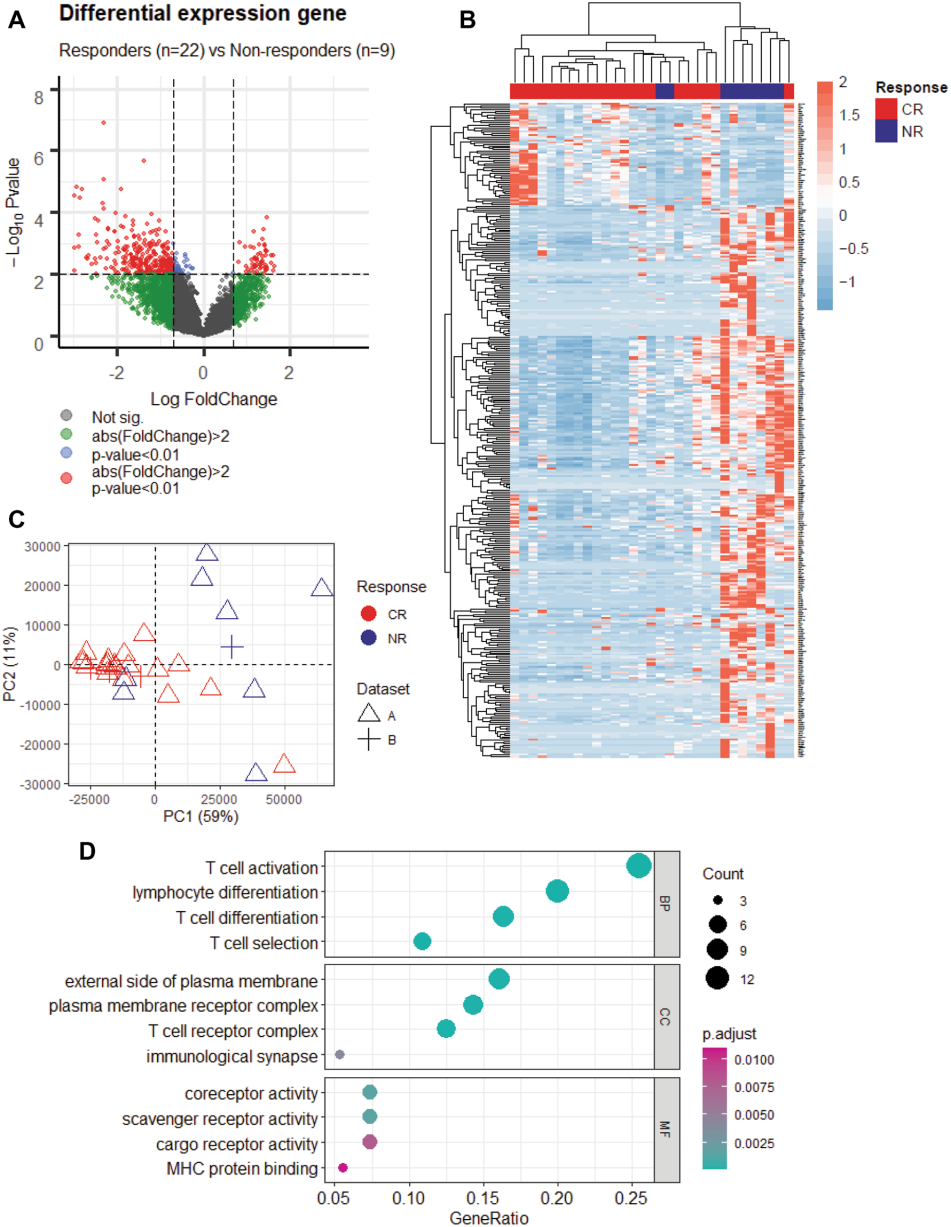
Differentially expressed genes mainly correlated with T cell activations. (**A**) Volcano plot of the 360 clinical remission-related DEGs. Fold Change > 2 & *p*-value < 0.01 were set as screening criteria. *DEGs* differentially expressed genes. Responders represent CR; Non-responders represent NR; (**B**) Unsupervised clustering heatmap of the differential expression genes between responders and non-responders; (**C**) Principal component analysis in CR and NR based on differentially expressed genes; (**D**) Gene oncology analysis for upregulated genes. *BP* biological process; *CC* cellular component; *MF* molecular function. Circle size means gene number involved in each term. Circle color means p-adjust value.

From the above PCA, we noticed that there was no clear separation of patients experiencing complete remission (CR) and no response (NR) based on the analysis of the entire set of genes (Fig. 1B). Consequently, we looked for genes differentially expressed between bone marrow from patients with CR and NR. We used limma package in R to calculate the statistically significant differentially expressed genes (DEGs) and identified 359 DEGs, including 57 upregulated genes and 302 downregulated genes (Fig. 2A and Table S2). Normalized expression data from these 359 genes was used to perform supervised hierarchical clustering which showed a clear separation between CR and NR samples (Fig. 2B). PCA of the differentially expressed genes confirmed that CR patient bone marrows were distinct from NR patient bone marrows based on DEGs (Fig. 2C). Furthermore, gene oncology (GO) analysis conducted using the DEGs showed genes upregulated in CR patient bone marrows were mainly involved in T cell activation and differentiation, while downregulated genes had no obvious relationship with T cell function (Fig. 2D, S1). These results indicate the status of T-cells from bone marrow in each patient could also contribute to the clinical remission.

### Bone marrow from responders and non-responder show distinct activity in T cell function related pathways and cell cycle checkpoint

**Figure 3:**
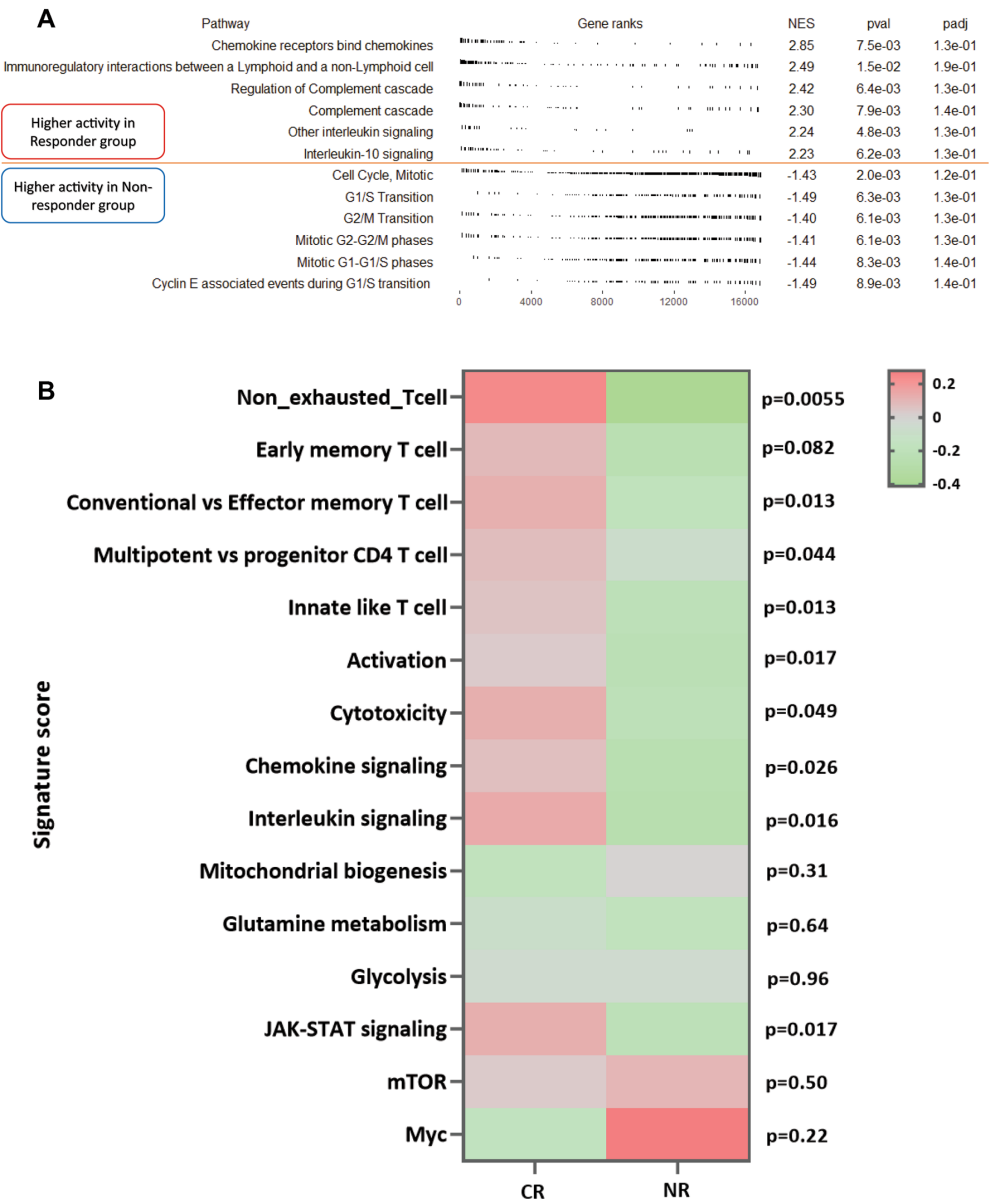
Different active pathway in responders and non-responders. (**A**) The top half above dash red line depicts activated pathways in the responder group and the bottom half depicts pathways activated in the non-responder group based on gene set enrichment analysis. (**B**) Heatmap shows different pathway activity score in CR and NR. Color represents *p*-value.

To further explore the genomic differences in CR and NR patient bone marrow, we conducted gene set enrichment analysis (GSEA) using expression data from all genes, not just the differentially expressed genes. As shown in Fig. 3A, chemokine and interleukin signaling pathway activity was higher in CR patient bone marrows while NR patient bone marrows showed greater cell cycle associated pathway activity. Since GSEA only shows enriched pathways in a predetermined set, gene set variation analysis (GSVA) was performed using custom pathways involved in T cell function. In addition to chemokine and interleukin signaling pathways, we also saw that CR patient bone marrows are enriched in gene expression profiles involved in early memory cell differentiation (Fig. 3B). These results suggested that the microenvironment of bone marrow affects clinical remission.

### Identification of a 14-gene signature as a biomarker for predicting clinical remission

**Figure 4:**
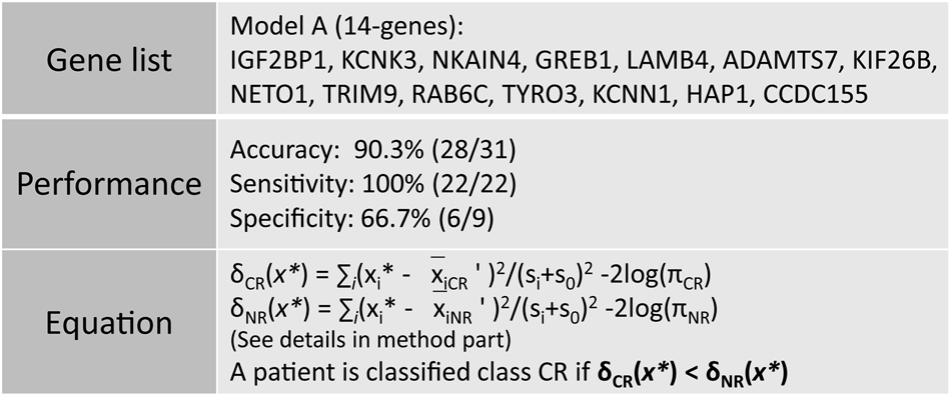
Development of prediction model to predict clinical remission. Prediction analysis for microarrays tool was used for prediction of clinical remission. The table includes 14 signatures’ symbol, performance from LOOCV and prediction equation of model. Detailed information of parameters in equation showed in method part.

To develop a classifier which can be used for predicting the clinical remission in ALL patients treated with CD19 CAR-T cells, we tried different machine learning methods in BRB_array tools. Finally, we developed a prediction model using prediction analysis for microarrays tool (PAM tool). In consideration of the relatively small number of patients in this study, we used a leave-one-out cross validation (LOOCV) method. A 14-gene signature was identified as a predictor of clinical outcome. Detailed information concerning this model including signature genes, performance, and equation are shown in Fig. 4. In this model, a patient is classified to the class CR if the δ_CR_(x*) < δ_NR_(x*), otherwise the patient is NR. The details of determining the discriminant score δ_CR_(x*) and δ_NR_(x*) are shown in the methods section. We found that the accuracy, sensitivity, and specificity of this model is 90.3%, 100%, and 66.7%, respectively.

## Discussion

This study explored candidate clinical remission-related genomic signatures in the bone marrow of patients with ALL prior to treatment with CD19 CAR T-cells. Our results show that chemokine, interleukin-related pathway activity was higher in the bone marrow microenvironment of patients who experienced a complete response and cell cycle checkpoint associated pathway activity was higher in patients who did not. Furthermore, we identified a 14-gene signature as a marker for predicting clinical remission following CD19 CAR T-cell immunotherapy in ALL patients.

Our study identified bone marrow biomarkers measured prior to CAR-T cell treatment that are associated with better clinical response. Other pre-treatment patient characteristics that affect the outcome of ALL patients treated with CAR T-cell include disease burden, high risk disease cytogenetics and disease molecular phenotype^8,21,22^.

Other biomarkers that are associated with the clinical outcome of CD19 CAR T-cell therapy have been described, but most involved CAR T-cells product characteristics or events occurring in the patient after the CD19 CAR T-cells have been administered^21,23^. Several studies have found that CD19 CAR T-cell expansion and persistence are associate with better clinical outcomes^12,22,24,25^. The expression of a more immature T cell phenotype such as Tscm phenotype by CD19 CAR T-cells has been associated with better clinical outcome^12,24^. CD19 CAR T-cells that have a mature phenotype and express immune checkpoints/exhaustion markers such as programed cell death protein-1 (PD-1), T cell immunoglobulin and mucin-domain containing-3 (TIMP-3) and lymphocyte activation gene-3(LAG-3) are less effective clinically^21^.

Response to immune therapies including CAR T-cells is also dependent on the tumor, the tumor stroma and immune cells within the tumor^26^. The immunosuppressive effective of tumor microenvironment has been well documented. Leukemic cells in the circulation and bone marrow in patients with ALL are likely much more accessibility to CAR T-cells than cancer cells in the tumor microenvironment, but our results suggest that the state of the bone marrow microenvironment is still important in the response of ALL patients to CAR-T cell therapy. Our study showed that a proinflammatory microenvironment may support effective CD19 CAR T-cell therapy.

The bone marrow microenvironment is important in protecting and maintaining both normal and leukemic stem cells^27^. In addition to blood, the bone marrow microenvironment contains endothelial cells, osteocytes, osteoblasts, adipocytes, mesenchymal stromal cells and macrophages. Some evidence suggests that monocytes are important in the bone marrow microenvironment. The qualities of non-classical monocytes are increased in the bone marrow from patients with ALL at the time of diagnosis while the number of classical monocytes is decreased^28^. Animal models have found that depleting non-classical monocytes from the marrow prolongs ALL remission following chemotherapy^28^. Blood biomarkers are also associated with CAR T-cell clinical outcome. Adults with lymphoma treated with CD19 CAR T-cells who had low levels of circulating myeloid derived suppressor cells (MDSC) pre- and post-treatment had better clinical responses. In this same group of patients those with markers a Th1 type of immune response also had better clinical outcomes^29^.

Other studies found that elevated lactate dehydrogenase (LDH) levels are associated with a less favorable outcome in patients with ALL who were treated with CAR T-cells. One study found that in adults with ALL who were treated with CD19 CAR T-cells lower pre-leukoreduction LDH levels and higher pre-leukocyte reduction platelet counts were associated with better event free survival^30^. The less favorable outcome in patients with high LDH levels and low platelet counts likely reflects more leukemia in the marrow and more rapidly progressive disease^30^. However, some studies suggest the elevated LDH levels are related to an immunosuppressive tumor microenvironment^21^.

The tumor microenvironment is also important in lymphoma patients who were treated with CAR T-cells. In patients with B cell lymphomas who responded to CD19 CAR T-cell therapy, the responding tumors showed infiltration of CAR T-cells and the presence for activated T-cells^31^. The quantities of activated T cells present were much greater than the quantities of CAR T-cells suggesting that T cell activation is important in clinical response^31^.

It is well known that pre-treatment leukocyte reduction improves clinical outcomes of CAR-T cell therapy^25^. Leukopenia results in increased CAR T-cell expansion which improves clinical outcome, however, leukocyte depletion may also affect immune cells within the bone marrow. Pre-clinical studies suggest that immune check point blockade improves CAR T-cell therapy for cancers. It is possible that the administrative to immune checkpoint inhibitors might improve the outcome of CD19 CAR T-cell therapy in ALL patients who do not have a bone marrow microenvironment with T cell activation pathways^32,33^.

In summary, our results indicate the combined effect of specific drugs activating chemokine, interleukin-related pathway and CAR T-cell immunotherapy may be beneficial in T cell activation and clinical remission. However, this analysis was only based on the limited accessible datasets and patients’ number. Further investigation is needed to collect and track more samples to confirm the findings.

## Supplementary Information

Supplementary Information 1.

Supplementary Information 2.

Supplementary Information 3.
