# Supplementary material for: Identification of genomic signatures in bone marrow associated with clinical response of CD19 CAR T-cell therapy
Source: Sci Rep. 2022 Feb 18;12:2830. doi: 10.1038/s41598-022-06830-3 (PMC8857276; doi:10.1038/s41598-022-06830-3)
Supplement: Supplementary file 1 — Supplementary Information 1. [file 41598_2022_6830_MOESM1_ESM.docx]

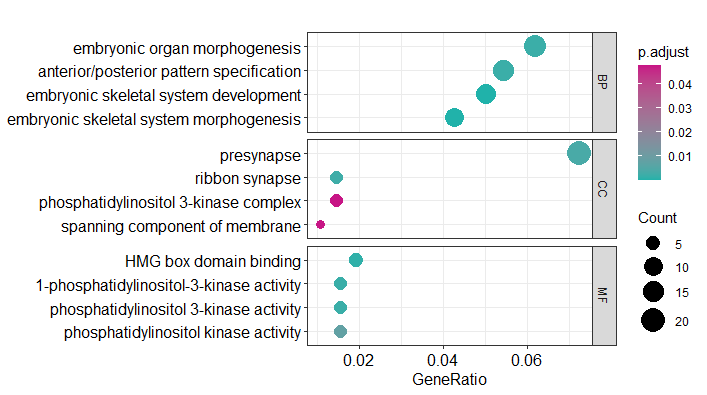


**Figure S1. Gene oncology analysis for upregulated genes.**

BP: biological process; CC: cellular component; MF: molecular function. Circle size means gene number involved in each term. Circle color means *p*-adjust value.
